# 2^nd^ Brazilian Symposium on Frontotemporal Lobar Degeneration / 2009

**Published:** 2009

## ABSTRACT–1

**Language evaluation in three patients with frontotemporal dementia associated with amyotrophic lateral sclerosis.**

Freitas MIA, Bahia VS, Mansur LL

Department of Neurology, Hospital das Clínicas, University of São Paulo School of Medicine, São Paulo, SP, Brazil.

***Background:*** The association of FTD (Frontotemporal Dementia) with ALS (Amyotrophic Lateral Sclerosis) may be underestimated by quickly progressive course of disease. Some researchers support the continuum hypothesis of both diseases. Language impairment may be one of symptoms that are characterized by reduced linguistic expression.

***Objectives:*** To analyse language performance of FTD/ALS patients.

***Methods:*** Three FTD/ALS patients (2 female and 1 male) were evaluated by Arizona Battery for Communication Disorders of Dementia. Two patients had ALS diagnosis and one patient had only prodromic signs but some of your relatives have the diagnosis and they are following.

***Results:*** Age mean of patients was 46 years and education mean was 12 years. The severity level and disease duration were respectively: CDR 1 – 12 months; CDR 1 – 24 months and CDR 3 – 36 months. One patient showed severe language deterioration and two patients showed episodic memory, linguistic expression and visuo-spatial impairments. Dysphagia and dysarthria were identified in two patients.

***Conclusions:*** Language impairments were most prominent in linguistic expression tasks, as was expected; however, episodic memory and visuo-spatial deficits were also verified.

## ABSTRACT–2

**Oral discourse in fronto-temporal lobar degen- eration.**

Toledo CM, Souza R, Mansur LL, Bahia VS

Department of Neurology, Hospital das Clínicas, University of São Paulo School of Medicine, São Paulo, Brazil.

Fronto-temporal lobar degeneration (FTLD) is considered the second most frequent cause of dementia, having three clinical manifestations: frontal-temporal dementia (FTD), semantic dementia (SD), and primary progressive aphasia (PPA). The study of the discourse offers information on various linguistic levels: phonological, syntactic, semantic and pragmatic, in which the patients with FTLD could present difficulties.

***Objective:*** To obtain references from oral descriptive discourse, in subjects with FTLD.

***Method:*** Four subjects diagnosed with FTLD (one with FTD, two with SD and one with PPA) with sixty years or more were evaluated. Four figures, each depicting a different scene (two simple and two complex figures) were used.

***Results:*** The results indicated great difficulty in producing the total propositions of the figure, which worsened in accordance with material complexity. The lesser the number of elements identified in the figure, the greater the difficulty of naming and monitoring.

***Conclusion:*** The results demonstrate difficulties in discourse organization, difficulties in selecting the most relevant items of the figure, formulation and monitoring mistakes in all patients of sub-groups.

***Key words:*** narrative, language evaluation, fronto-temporal lobar degeneration.

## ABSTRACT–3

**Age-related differences in speech monitoring.**

Steiner VS, Mansur LL

Department of Neurology, Hospital das Clínicas, University of São Paulo School of Medicine, São Paulo, SP, Brazil.

Speech monitoring is very important in speech production to assure correct speech output. It requires an executive control to detect and repair the errors produced. Since some executive processes may be impaired in the healthy elderly, the aim of this study is to investigate age effect on speech monitoring.

***Objectives:*** To analyze adult age differences in error detection and self-repair. The generated data were correlated with verbal fluency performance.

***Methods:*** The monitoring abilities were evaluated according to the most widely accepted monitoring theory. Forty-eight healthy subjects divided in four groups of age ranging from 30 to 80 years described visual networks and were asked to detect and self-correct the errors they produced.

***Results:*** A significant difference (p= .05) among the groups was found relative to the total number of errors produced while no significant difference (p= .35) was found in the monitor’s accuracy (percentage of self-repaired errors over total number of errors produced).

***Discussion:*** Although the elderly produced nearly three times more errors then the young adults, they are not different from them in speech monitoring mechanisms because they detect and repair the same percentage of errors when compared to the young. Linguistic and cognitive factors contribute to efficient per formance in speech monitoring in normal aging. Future studies should attempt targeting subjects with executive decline.

## ABSTRACT–4

**Semantic tests in normals: Pyramids and Palm Trees and Kissing and Dancing.**

Mansur LL, Barbieri MBP, Carthery-Goulart MT

Department of Neurology, Hospital das Clínicas, University of São Paulo School of Medicine, São Paulo, SP, Brazil.

The objective of the Pyramids and Palm Trees test (PPT) and Kissing and Dancing (KD) is to evaluate the capacity to access detailed semantic representations from figures and words. The two tests consists of 52 triads of items of objects (PPT) and verbs (KD) in which one stimulus is located at the top of the page and two at the bottom. The two items at the bottom are semantic correlated while the one at the top is from a different category. The choice should be made based on some property or association shared with the top item.

***Objective:*** To verify the applicability of the two tests in young healthy Brazilian population.

***Method:*** We examined young healthy Brazilians. Results: 22 young Brazilians, 20 female and 2 male, mean age of 21.81 (SD=1.36) performed the test. The median score of the group in PPT was 46.34 (89.11%) and 47.38 (91.11%) in the KD. In PPT 14 figures obtained less than 90% of correct choices, with one of them only 4 (18%) correct. In KD, 16 figures obtained less than 90% of correct choices.

***Discussion:*** The median of correct answers in PPT and KD were similar to the normal subjects of this sample. Both PPT and KD need adjustments that respect socio cultural peculiarities in order to meet the evaluation proposal in Brazilian population.

***Key words:*** semantic system, language assessment, cognition.

## ABSTRACT–5

**Evolution of artistic manifestation in degenerative disease with predominant compromise in parietal lobes. Case report.**

Mansur LL, Nitrini R 

Department of Neurology, Hospital das Clínicas, University of São Paulo School of Medicine, São Paulo, SP, Brazil.

There are reports in literature of individuals with dementia, specially lobar frontotemporal degeneration complex, with language alterations, who discovered possibility of expresssion through visual arts, such as painting. Reports of cases where individuals developed artistic skills prior to manifesting degenerative disease are rare.

***Objective:*** To compare changes in artistic production, before and after the onset of parietal degeneration diagnosed as cortico-basal degeneration (CBD).

***Methods:*** Twelve paintings were digitalized, 5 produced before manifestation and five after the disease in a male patient who was diagnosed at the age of 55. The images represent a sequence of production during a period of approximately 5 years (1999–2004).

***Results:*** It was possible to affirm that, during this time, there was a change in the focus of representation, through evolution of sketches (traces) that, in the context of severe ideomotor apraxic difficulties, he was distanced from a figurative referencial, and moved to explore color and shape. One of the interesting features of this group of artwork is preservation, throughout the process, of a harmonic and balanced sense of esthetics. The artistic skills in this individual with CBD were maintained and transformed utilizing vehicles demanding praxic skills with a lesser degree of precision. In the case study it is impossible to attribute a negative connotation of loss to the evolution observed.

***Key words:*** art, creativity, parietal lobe, corticobasal degeneration.

## ABSTRACT–6

**Case study of foreign accent syndrome.**

Biudes F, Almeida PR, Mendonça LIZ, Mansur LL

Department of Neurology, Hospital das Clínicas, University of São Paulo School of Medicine, São Paulo, SP, Brazil.

Foreign accent syndrome is described as the result of a vascular lesion however there is a report of its presentation as the first sign of primary progressive aphasia.

***Objective:*** To describe and characterize foreign accent syndrome in the context of other language alterations resulting from vascular conditions.

***Methods:*** Study of a female patient, 59 years of age, right-handed, speaks Brazilian Portuguese exclusively. She suffered an ischemic stroke in the left-parietal region, in 2006. Some sequela remained, and her main complaint was foreign accent. The neurological exam revealed the absence of sensorimotor alterations and hemianopsia; auditory and vision were functional. The language evaluation indicated compromise of comprehension of commands (5/10=50%) and complex material (4/6-67%); deficits in repetition of words (3/5=75%) and phrases (60%), responsive naming (6/10=60%) and visual confrontation (5/15=33.3%). In the word and non-word reading there was an alteration in syllable stress and phoneme pronunciations when the graphemic conversion in Portuguese is irregular. Spontaneous speech revealed traces of agrammatism; 7.6 minutes of spontaneous speech, reading and counting were analyzed using Praat software. Stress alterations were noted with reference to Brazilian Portuguese (33 episodes), 5 omissions; 23 vowel and consonant substitutions and prosodic intonation elements that differ from standard Brazilian Portuguese, in other words, beyond the stress variation, from an acoustic spectrum diversity was also observed in the pitch curves.

***Discussion:*** The foreign accent was observed in automatic and voluntary tasks as well as in isolated situations or complex language processing. It occurred in rhythm and melodic prosodic components and was not exclusively conditioned by the agrammatism.

## ABSTRACT–7

**Rapidly progressive anarthria as a manifestation of ftld -mnd: association with semantic dementia?**

Takada LT, Brucki SMD, Silagi ML, Senaha MLH, Grinberg LT, Mansur LL, Nitrini R 

Department of Neurology, Hospital das Clínicas, University of São Paulo School of Medicine, São Paulo, SP, Brazil.

***Background:*** The link between frontotemporal lobar degeneration (FTLD) and motor neuron disease (MND) has been grounded on clinical and neuropathological associations, and FTLD-MND is currently labeled as a TDP-43 proteinopathy.

***Objectives:*** To describe a case of FTLD-MND.

***Case report:*** A 59 years old male patient presented with a history of rapidly progressive anarthria in the previous five months and a three-month history of gait difficulties, coupled with comprehension deficits. Neurologic examination showed pyramidal syndrome manifested as spastic bilateral hemiparesis. Lower motor neuron signs appeared two months after the initial evaluation, with amyotrophy and widespread fasciculations, thus establishing a clinical diagnosis of amyotrophic lateral sclerosis. Neuroimaging studies showed bilateral anterior temporal lobe atrophy, more prominent on the left side. A left temporal biopsy was performed, which revealed ubiquitin positive cytoplasmatic inclusions. Immunostaining for tau, beta-amyloid and alfa-synuclein was negative. The patient died about seven months after the beginning of the symptoms.

***Conclusions:*** In this case, MND was combined with neuroimaging findings usually observed in Semantic Dementia (SD). The neuropathological diagnosis was of FTLD with ubiquitin-immunoreactive inclusions (FTLD-U). We discuss the challenge of examining semantic memory in an anarthric patient and the rarity of the association between MND and SD.

## ABSTRACT–8

Caregiver burden in dementia with and without behavioral symptoms.

Lopes DB, Reis GDS, Caixeta L

Federal University of Goiás, GO, Brazil.

***Background:*** Behavioral and psychological dementia symptoms have generally been thought to be of secondary importance, but new evidence suggests that these are important determinants of patients’ distress, caregiver burden, and outcome in dementia.

***Objectives:*** To compare the level of anxiety and depression between the caregivers of dementia patients that present behavioral alterations and patients that don’t.

***Methods:*** The participants were caregivers of 40 dementia patients served in the dementia outpatient division. A semi-structured questionnaire was used to know about sociodemographics caregiver’s dates, about the cares of them with the patients and the kind of dementia. The Hamilton’s scale of anxiety and depression were used to assess the affective disorders in these caregivers.

***Results:*** The majority of the caregivers in both groups was female gender, married, has a familiar degree and presented with serious levels of anxiety and depression. The most prevalent dementia forms on the group of behavioral alterations were FTD and infectious dementia, while on the other group was the parkinsonism plus. There were differences about anxiety and depression levels among the caregivers of dementia patients with behavioral alterations when compared with those without.

***Conclusions:*** Caregiver burden is very common in dementia with and without behavioral symptoms.

## ABSTRACT–9

**Performance of fltd patients in an artistic task – a pilot study.**

Anauate MC, Bahia VS, Nitrini R, Chiesa R, Radanovic M

Department of Neurology, Hospital das Clínicas, University of São Paulo School of Medicine, São Paulo, SP, Brazil.

***Background:*** Several studies have addressed visuospatials skills in artistic activities in early to moderate stages FTLD, despite the behavioral impaiment and executive dysfunction. Miller et al. (2005) studied the emergence of artistic abilities in FTLD, Rankin et al. (2007) published the first study with a quantitative method to evaluate art production in FTLD.

***Objectives:*** To investigate the performance of subtypes of FTLD (SD, PPA and FTD), compared to controls in visuospatial and artistic tasks.

***Methods:*** 6 FTLD patients (age: 67.3±19, schooling: 13.2±10.4) and 3 controls (age: 56.3±8.9, schooling: 15.5±8.3) were assessed by a 3-stage artistic protocol: visual observation of a Sisley’s painting, copy, and a collage based on the same picture.

***Results:*** FTLD patients had lower scores than controls in Picture Observation and Description (3±0/4±0),Visuospatial Perception (13±3/20±0), Copy (16.3±4.5/20.6±3.5), Collage (20.1±5/24.6±3), Examiner’s Observation (21.1±4.5/24±0), and Total (70.5±11/89.3±6.5).

***Conclusions:*** FTLD patients presented impairment in visuospatial and executive skills required to perform artistic tasks (visual recognition, picture representation, and spatial planning). The structured study of artistic abilities may be a useful alternative tool in the assessment of cognitive dysfunction in FTLD patients, in which traditional neuropsychological testing may prove difficult due to behavioral disturbances.

## ABSTRACT–10

**Insight deficits (anosognosia) in frontotemporal dementia and Alzheimer’s disease.**

Caixeta LF^1^, Nitrini R^2^

^1^Federal University of Goiás, GO, Brazil. ^2^Department of Neurology, Hospital das Clínicas, University of São Paulo School of Medicine, São Paulo, SP, Brazil.

***Background:*** We objective to describe and compare in sight deficits and related variables in Alzheimer’s disease (AD) and frontotemporal dementia (DFT) in order to suggest potential neurobiological basis to self-awareness and a particular affiliation to frontal lobes.

***Methods: ***Twenty patients with FTD and 22 with AD were evaluated in one specific dimension of self-consciousness: insight. Cognitive, psychopathological (Frontal Behavioral Inventory) and neuroimaging measures were statistically analyzed in association with measures of insight.

***Results:*** Insight deficits were invariably important and frequent in FTD, but more mild and variable in AD. Insight deficits showed association with frontal damage. Insight was significantly associated with some variables (severity of illness, functional adaptation, negative symptoms), but not with others (sex, age, duration of illness, years of education) in a general form for FTD and AD groups.

***Conclusions:*** Insight deficits seem to be more related to the topography of degenerative lesions than to the type of dementia and have predictive value to program more early and intensive actions in order to minimize socio-functional limitations related to deficits in insight. Such deficits seem to constitute markers of the pathophysiological process involved in the deterioration of the frontal lobe executive functions. Many of the more representative FTD behaviors may be understood as phenomenological manifestations related to insight deficits.

## ABSTRACT–11

**Repetitive and compulsive behaviours in frontotemporal dementia.**

Carmo RF, Caixeta LF, Reis G, Soares LD, Soares CD

Federal University of Goiás, GO, Brazil.

***Background: ***Patients with FTD typically have early noncognitive behavioral changes with relatively spared cognition, frontal atrophy and enlargement of the Sylvian fissures on CT and MRI scans, and frontal-temporal deficits on SPECT or PET scans. Compulsive behaviors frequently occur in FTD.

***Objectives:*** To document the prevalence and pattern of repetitive behaviour in patients with frontal and temporal variants of frontotemporal dementia. Secondly, to examine the relationship between repetitive and neuroanatomical data.

***Methods:*** Patients with the following were studied (fvFTD, n=8, semantic dementia, n=1), using the Neuropsychiatric Inventory (NPI), the Mini-Mental State Examination, neuroimaging (MRI and SPECT), and the Clinical Dementia Rating scale. Patients were rated on the devised Stereotypic and Ritualistic Behaviour (SRB) subscale, which was designed as an addendum to the NPI.

***Results:*** The SRB was significantly high in almost all patients. There is some degree of laterality (more left atrophy) and basal ganglia atrophy in most cases.

***Conclusions:*** Complex stereotypic behaviours are a core feature of the dementing syndrome in FTD and may reflect early and specific deficits in orbitofrontal circuitry and basal ganglia involvement.

## ABSTRACT–12

**Polar disorder x frontotemporal dementia (FTD) – report of case.**

Novaretti TMS

UNESP/Universidade Estadual Paulista, SP, Brazil.

***Introduction:*** The clinical diagnosis of FTD refers to a group of progressive overlapping clinical syndromes characterized by the insidious onset of behavioral changes, loss of word and object knowledge, and aphasia. FTD is a common cause of dementia in patients who are younger than 65, and in patients who are less than 60 years of age, FTD may be more common then AD.

***Report of case:*** LOD, 49 years, was brought the neurologist in June of 2002, with history of depression since 1996. He was submitted to many treatments without success. He worsened in 1999, when he began to speak in killing the family. Although he resided in a small city and was married, he arranged a girlfriend and went go for a walk with her at the central city square. He didn’t get to understand why the wife asked for separation after this incident. He never had presented previously other depressive episode. There was not depression among his relatives. Examination: obese, MMSE=22 (patient with 12 years of literality).HD: FTD. His MR was normal and SPECT revealed “Moderate hipoperfusion of the frontal lobes and anterior portion of the temporal lobes”-14/01/2003. He was treated with Risperidone and Clonazepan. and presented improvement of behavior. In 2006, the diagnosis was questioned because MMSE was repeated and the punctuation was 30. New SPECT showed improvement. The patient continue stable since 2003.

***Conclusion:*** The Maniac episode of Bipolar Disorder may be precipitate when antidepressives are prescript without stabilizers of the humor. The improvement of SPECT makes us to suppose that is a functional disease, not FTD.

## ABSTRACT–13

**Performance in tests of executive functions among patient with refractory schizophrenia and frontotemporal dementia.**

Viana RM, Bahia VS, Elkis H, Nitrini R

Hospital das Clínicas, University of São Paulo School of Medicine, São Paulo, SP, Brazil.

***Backgrond:*** The refractory schizophrenia is characterized by no response to the treatment in appropriate doses and periods with two antipsychotic, and they represent 30 to 60% of the cases. The frontotemporal lobar degeneration (FTLD) is the second most common form of presenil degenerative dementia. There are evidences that cognitive declines in schizophrenia can resemble FTLD deficits, and presence of deliriums, hallucinations and psychotic symptoms are mentioned in some FTLD case reports. In FTLD the executive functions (EF) are usually affected. Several studies have been showing that EF has almost always been associated to negative symptoms of schizophrenia as well and responses poorly to drugs.

***Objectives:*** To compare performance of the subjects (FTLD and Schizophrenics) in tests of EF, to compare EF tests performance with the performance in tasks of the daily life, and also with presence and intensity of behavioral alterations.

***Methods:*** 20 patients with diagnosis of refractory schizophrenia and 20 patients with FTLD will be selected. The subjects will be submitted to a neuropsychological battery, evaluating planning, flexibility, motor performance, working memory, inhibitory control, abstraction. Relatives will answer to Neuropsychiatric Inventory (NPI) and the Brazilian version of the Disability Assessment for Dementia (DAD-Br).

## ABSTRACT–14

**The contribution of single photon emission computed tomography with statistical parametric mapping in the differential diagnosis of frontotemporal dementia and Alzheimer disease.**

MS Mari-Nilva, Viana R, Porto CS, Bahia VS, Nitrini R 

Hospital das Clínicas, University of São Paulo School of Medicine, São Paulo, SP, Brazil.

***Background:*** Alzheimer disease (AD) and Frontotemporal Dementia (FTD) are the two leading degenerative causes of dementia, especially in presenile years. Distinction between them is sometimes challenging due to clinical and even neuroimaging overlapping. A correct diagnosis is paramount as it has consequence on the therapeutic management and AD specific treatment is available. Furthermore understanding on pathophysiology of FTD is increasing and therapeutic trials have been envisaged. Single Photon Emission Tomography (SPECT) can show changing in regional perfusion indicating an underlying pathological process even when no structural modification is detectable. Patients with AD often have reduced blood flow in the temporoparietal regions in contrast with frontotemporal hypoperfusion in FTD. SPECT has been used in the differential diagnosis of FTD and AD yielding an accuracy of up to 87% (Bonte et al., 2006). When Statistical Parametric Mapping (SPM), a voxel-based analysis method witch compare data from patients to healthy volunteers, is applied to SPECT, it can result in a greater sensitivity (Kemp, 2005). Direct comparison of AD and FTD using SPECT-SPM has been done only once highlighting some networks impaired more specifically in either disorder (Varrone et al.,, 2002).

***Objectives:*** To evaluate the contribution of SPECT with SPM on the differential diagnosis of AD and FTD.

***Methods:*** 40 subjects fulfilling the NINDS-ADRDA criteria for probable AD and 20 patients with FTD according to current clinical criteria (Neary et al., 1998) and 40 healthy volunteers. Diagnosis will be established in a consensus meeting and considered positive only the cases whose diagnosis remains unchanged after one year. All subjects will undergo brain CT scan or MRI, blood tests to rule out other causes and a complete neuropsychological evaluation, including tests for memory, executive functions, language, visuo-spatial abilities, behave and functional status.

***Results:*** There is no result available from this ongoing work yet.

## ABSTRACT–15

**Comparative evaluation of subcortical atrophy in frontotemporal dementia and dementia with extrapyramidal signs.**

Vieira RT, Caixeta L

Federal University of Goiás, GO, Brazil.

***Background:*** Cerebral subcortical atrophy rate is a biomarker for disease progression in neurodegenerative disorders including Frontotemporal Dementia (FTD) and other types of dementia, but its significance for clinical manifestations and the relationship with Extrapyramidal Signs (EPS) have not been extensively investigated.

***Objectives:*** To compare the severity of cerebral subcortical atrophy in FTD and dementia with extrapyramidal signs, and to analyze the correlation between cerebral subcortical atrophy and demographic and clinical characteristics at both groups.

***Methods:*** Nineteen FTD patients without EPS and twenty-one patients with EPS in dementia (6 Vascular Dementia, 5 Alzheimer’s disease, 4 FTD, 4 Parkinson Disease Dementia, 2 Lewy Body dementia) formed the sample, which comprised 19 men and 21 women, aged 33 to 99, with mean age (±SD) of 68.35±13.09, with schooling ranging from 1 to 20 years, with mean (±SD) of 8.06±6.52, and disease duration with a mean of 3.84±3.44. The degree of cerebral subcortical atrophy was measured indirectly with a linear measurement of subcortical atrophy, the Bifrontal Index (BFI), using magnetic resonance imaging. We evaluated cognition, activities of daily living and dementia severity with the Mini-Mental State Examination, Functional Activities Questionnaire and the Clinical Dementia Rating, respectively.

***Results:*** There was no significant difference (p=0.371) in BFI between FTD and the group with EPS. The group with dementia and EPS exhibited statistically higher age than FTD group (p=0.01). The severity of cognitive deficits for both groups and dementia severity (only for EPS in dementia group) were correlated with BFI.

***Conclusions:*** A linear measurement of cerebral subcortical atrophy did not differentiate FTD from EPS in dementia group in this sample. Cognitive function (at both groups) was inversely correlated with cerebral subcortical atrophy and the presence of EPS suggested association between cerebral atrophy rate and severity of dementia.

## ABSTRACT–16

**Profile of demented patients in out-patient tertiary hospital.**

Varandas PRBR, Suemoto CK, Mansur LL, Morillo LS

Hospital das Clínicas, University of São Paulo School of Medicine, São Paulo, SP, Brazil.

***Introduction:*** The ageing of the population conditions an increase in the prevalence of dementia, where 50% correspond to moderate and severe phases. Nonetheless, the profile of these patients remains poorly-characterized, making management difficult.

***Objective:*** To describe the clinical characteristics of the moderately and severely demented (CDR 2 and 3) of a university hospital out-patient clinic in the city of São Paulo. A descriptive analysis of the socio-demographic, clinical, cognitive, functional and behavioral profiles and the type and duration of medications specific for dementia was realized.

***Results:*** 70% female, age 82.06±5.2 years, schooling 4.08±3.3 years, 65% Alzheimer Disease, 23.3% Cerebrovascular Dementia associated to Alzheimer Disease, 8.3% Cerebrovascular Dementia, 3.4% other diagnoses. 4.5 co-morbidities, Mini Mental 9.73±6.6, Mini Mental severe 12.85±7.5, 55% CDR-2, INP 25.51±22.3, the following items being more common: apathy, aberrant motor behavior, nocturnal anxiety and behaviors. 90% take anti-cholinesterase (medium 2.19 years of use), 46.7% take anti-psychotic medication.

***Conclusions:*** This is a very aged, female population with low levels of schooling, multiple co-morbidities, and a greater prevalence of degenerative disease. Although 50% of the patients take anti-psychotics, the psychotic symptoms were infrequent in relation to the other items of INP.

## ABSTRACT–17

**Neuropsychiatric symptoms in moderate and severe dementia: is there a difference?**

Suemoto CK, Varandas PRBR, Mansur LL, Morillo LS

Hospital das Clínicas, University of São Paulo School of Medicine, São Paulo, SP, Brazil.

***Introduction:*** Neuropsychiatric symptoms (NPS) in dementia are common during the evolution of the disease. They are associated to greater wear of the proxy, cognitive decline and premature institutionalization. Nonetheless, data about NPS in more advanced stages of dementia are rare.

***Objective:*** To compare NPS in patients with moderate and severe dementia.

***Methods:*** 60 out-patients of a tertiary hospital were classified as moderately (CDR 2) and severely (CDR 3) demented. The groups were compared relative to their scores on the NPI (Neuropsychiatric Inventory) scale and its 12 items.

***Results:*** The NPI score was similar between the groups (p=0.326). However, when the NPI items were analyzed, a difference between the groups was found in agitation (p=0.048), euphoria (0.024) and appetite alteration (0.041). Those with severe dementia scored higher on these items relative to those with moderate dementia. The groups were similar in relation to delusions, hallucinations, depression, anxiety, apathy, disinhibition, irritability, aberrant motor behavior and nocturnal behavior.

***Conclusion:*** Although the total NPI scores of moderate and severe dementia are similar, some items show differences between groups. These findings contributes to understand this numerous, but yet little studied population.

## ABSTRACT–18

**The discourse and narratives in the bipolar disorder experience.**

Neto R, Almeida ML, Anjos EU 

Federal University of Ceará, CE, Brazil.

***Background:*** Narratives of sickness need more human answers, since they translate the ideas of time and space of life history related to pain and suffering, therefore, this work evaluates the importance of them, especially the ones on depression.

***Objectives***: Understand the behavioral and temperamental processes involved in bipolar disorder, through the personal and family history reported in narratives of patients from the rural zone, at a basic health care unit, taking into account bipolar disorder diagnosis.

***Methods:*** The study sample consisted of 30 patients with ages varying from 12 to 67 years, referred by a Family Health Program team from the rural municipality of Aparecida – PB, diagnosed with Bipolar Disorder (DSM-IV).

***Results:*** Of the 30 patients interviewed, 21 attained the parameters established for bipolar disorder, with a predominance of depression associated to moderate/serious levels of euphoria, of these 11 had indicators of a family history of disorders. Twelve patients had difficulty in determining the onset of their disorder, citing various terminologies contained in the diagnosis, which indicated an imprecise diagnosis at the first visit.

***Conclusion:*** Through the identification of bipolar disorder, patients lack a translation of the different manners mobilized by behavioral and temperamental processes in the translation of the harm done to their daily lives.

## ABSTRACT–19

**Neuropsychological profile of patients with frontotemporal dementia.**

Nery M, Caixeta L, Soares VLD, Soares CD

Federal University of Goiás, GO, Brazil.

***Background:*** Recent work has clarified the neuropsychological features that distinguish fvFTD from Alzheimer’s disease, but few works has been conducted in Brazil in order to adress cognitive presentation based on our particular cultural background.

***Objectives:*** Verify the neuropsychological profile of brazilian’s patients with frontotemporal dementia, looking for particular aspects of this population.

***Methods:*** We analyzed 30 new patients with frontotemporal dementia, primary progressive aphasia and semantic dementia diagnosed according Neary et al. criteria selected from a consecutive series of our outpatients as to the details of neuropsychological symptoms.

***Results:*** With regard to central aspects of cognitive processing, such as memory, patients with fvFTD may have a distinctive profile of performance on tests of explicit and implicit memory, and contrary to current views some patients may even have a severe amnesic syndrome. Despite relatively well preserved general language skills, patients with fvFTD have particular problems with verb processing.

***Conclusions:*** Neuropsychological deficits in FTD may help to elucidade some of the aspects of underlying deficits in social judgement, theory of mind, processing of emotional stimuli and decision making. Frontotemporal dementia remains a powerful model for studying brain-behavior relationships.

## ABSTRACT–20

**Creativity in frontotemporal dementia.**

Souza LC^1,2,3*^, Volle E^1,2,3,4*^, Bertoux M^1,2,3^, Czernecki V^1,2,3^, Funkiewiez A^1,2,5^, Allali G^1,2,3^, Sarazin M^1,2,3^, Habert M-O^7^, Dubois B^1,2,3,5^, Kas A^7^, Lévy R^1,2,3,8^

^1^Université Pierre et Marie Curie-Paris 6, Centre de Recherche de l’Institut du Cerveau et de la Moelle Epinière, UMR-S975, Paris F-75013, France

^2^Inserm, U975, Paris, F-75013, France

^3^Cnrs, UMR 7225, Paris, France

^4^Institute of Cognitive Neuroscience - UCL (University College London) – UK

^5^AP-HP, Groupe Hospitalier Pitié-Salpétrière, Fédération de Neurologie, Paris, F-75013, France

^6^Geneva University Hospitals and University of Geneva, Geneva, Switzerland

^7^AP-HP, Groupe hospitalier Pitié-Salpétrière, Service de Médecine Nucléaire, Paris, F-75013, France

^8^AP-HP, Hôpital Saint-Antoine, Paris, F-75012, France.

***Background:*** The prefrontal cortex is thought to play a critical role in creative thinking. It is thus expected that severe damage to the prefrontal cortex leads to poor creativity. However, previous works showed the emergence of artistic talent (“increased creativity”) in FTLD patients.

***Objectives:*** To verify if: 1) creativity is increased or impaired after frontal degeneration, 2) poor creativity is associated with frontal dysfunctions and, 3) creativity production in FTLD is related to specific regional brain hypoperfusions.

***Methods:*** 17 fvFTLD patients and 17 matched controls performed the Torrance Test of Creative Thinking (TTCT) and a battery of tests assessing frontal functions. Brain SPECT was also performed in patients and correlated to the performance in the TTCT.

***Results:*** fvFTLD patients were strongly impaired in all dimensions of the TTCT. Disinhibited and perseverative responses were observed in several patients. Poor creativity was positively correlated with several cognitive and behavioural frontal tests. Poor creativity was also correlated with prefrontal hypoperfusion.

***Conclusions:*** fvFTLD is associated with poor creativity. The anatomical and functional integrity of the prefrontal cortex is strongly associated with creative thinking. The emergence of artistic talent in fvFTLD may be explained by the release of involuntary behaviours rather than by the development of creative thinking.

## ABSTRACT–21

**Effect of semantic priming in a task of lexical decision in two cases of degeneration lobar frontotemporal.**

Salles JF^1^, Holderbaum CS^2^, Mansur LL^3^

^1^Professora Adjunta do Instituto de Psicologia, PPG em Psicologia da Universidade Federal do Rio Grande do Sul - UFRGS, Porto Alegre RS, Brazil;

^2^Doutoranda do PPG em Psicologia da Universidade Federal do Rio Grande do Sul - UFRGS, Porto Alegre RS, Brazil;

^3^Department of Neurology, Hospital das Clínicas, University of São Paulo School of Medicine, São Paulo, SP, Brazil.

In the paradigm of semantic priming, a semantic context is provided with the presentation of an isolated word (prime), followed by the presentation of a series of letters (target).

***Objective:*** To investigate access to lexical-semantic information, through the paradigm of semantic priming, in two patients diagnosed with lobar frontal-temporal dementia.

***Methods:*** **Case 1** – 77 years of age, female, 11 years schooling, neuro-image with bilateral widening and symmetric frontal-parietal subarachnoid, associated to accentuation of cortical grooves and fissures, diagnosed with primary progressive aphasia, onset of 20 months. **Case 2** – 67 years of age, female, 11 years schooling, neuro-image with discrete dilatation of supra-tentorial ventricular system. Prominent peri-vascular spaces, atrophy anterior and inferior, temporal, diagnosed with semantic dementia, onset 48 months. The patients were requested to realize a task of lexical decision as quickly and accurately as possible. Half of the targets were real words (50% were preceded by semantically related primes) and the other half by pseudo-words.

***Results:*** In semantic priming Case 1 performed precisely. Case 2 was very imprecise responding to the pseudo-words (36% errors). Case 1 presented, on average, answers more quickly (<TR) than case 2. Although the reaction time for both patients had been less for targets preceded by related primes, considering the condition of primes not-related to the target, the difference was not statistically significant. For answers to targets preceded by related primes, none of the patients demonstrated any benefit of semantic context, in other words, there was neither greater precision nor speed in answering.

***Conclusion:*** In these two cases, lexical-semantic information, even when evaluated implicitly, was compromised.

